# Cost Effectiveness of a Multidisciplinary Perioperative Protocol for High‐Risk Emergency Major Abdominal Surgery in a Regional Victorian Hospital

**DOI:** 10.1111/ans.70299

**Published:** 2025-08-22

**Authors:** Jason Douglas Cox, Ayooluwatomiwa I. Oloruntoba, Kate Booth, Travis Murphy, Cameron Knott, Chun Hin Angus Lee

**Affiliations:** ^1^ Bendigo Health, Monash University School of Rural Health Bendigo Victoria Australia; ^2^ Alfred Health, Monash University Melbourne Victoria Australia; ^3^ Bendigo Health Bendigo Victoria Australia

**Keywords:** cost‐effectiveness analysis, emergency surgery, general surgery, quality improvementabdominal surgery

## Abstract

**Background:**

The Australian and New Zealand Emergency Laparotomy Audit (ANZELA) was implemented as standard‐of‐care for Emergency Major Abdominal Surgery (EMAS) at Bendigo Health in September 2021. All EMAS patients undergo routine preoperative risk assessment (RPRA) using the ‘National Emergency Laparotomy Audit (NELA) Calculator.’ Patients with NELA mortality risk estimates ≥ 10% are considered ‘high‐risk’ and routinely referred to the Intensive Care Unit (ICU) for planned postoperative admission. This study aims to identify whether RPRA and routine ICU referral for high‐risk EMAS patients is a cost‐effective intervention that improves postoperative outcomes in a rural health service.

**Methods:**

This study is a retrospective audit of high‐risk adult patients who underwent EMAS at Bendigo Health between September 2017 and August 2023. Postoperative outcomes and costs were compared before and after implementation of ANZELA. A cost‐effectiveness analysis was subsequently conducted to estimate the additional cost required to improve postoperative outcomes and presented as incremental cost‐effectiveness ratios (ICERs).

**Results:**

A total of 191 high‐risk EMAS patients were identified. The mean postoperative cost of care was AUD$52 338.78, with no significant change post‐ANZELA (*p* = 0.983). Post‐ANZELA, there was a 15.3% reduction in the rate of planned ICU admissions (*p* = 0.026), a 10.9% reduction in the rate of unplanned returns to theatre (*p* = 0.045), and a 16.83% reduction in the rate of severe postoperative complications (*p* = 0.03). There was no significant change in postoperative mortality (*p* = 0.59).

**Conclusion:**

RPRA and routine ICU referral of high‐risk EMAS improve perioperative outcomes without increasing cost‐of‐care. This protocol may therefore be a cost‐effective tool to guide the perioperative care of EMAS patients.

## Graphical abstract

Postoperative Cost of Care for High‐risk Emergency Major Abdominal Surgery pre and post ANZELA.

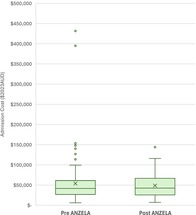

## 1 Introduction

The Australian and New Zealand Emergency Laparotomy Audit (ANZELA) was established to reduce the mortality associated with Australian emergency major abdominal surgery (EMAS) by identifying high‐risk patients and prompting implementation of risk mitigation strategies. The multidisciplinary perioperative protocol includes routine preoperative risk assessment (RPRA) for all EMAS cases using the ‘National Emergency Laparotomy Audit (NELA) calculator’, and classifies those with a NELA score 10% or greater as ‘high‐risk’ [1]. ANZELA recommends high‐risk patients are routinely admitted to an intensive care unit (ICU) for planned postoperative admission. Planned ICU admission for high‐risk EMAS patients lowers unplanned returns to theatre and unplanned escalations of care [2], likely facilitating earlier recognition and treatment of postoperative complications, thus preventing patient deterioration and ‘failure to rescue’ complications [1, 2, 3].

EMAS is expensive, costing the Australian public hospital system an estimated $AUD400 million per annum [4]. ICU beds are also a finite and expensive resource, with an average daily cost between $966 and $5381 [5]. Admitting EMAS patients to ICU for low‐value care would have downstream opportunity costs to the healthcare system as a whole. While multidisciplinary perioperative protocols which include RPRA and planned ICU admission for high‐risk EMAS patients have been shown to improve postoperative morbidity [2, 3] and mortality [6], there are limited studies exploring the cost effectiveness of these protocols. One UK study demonstrated implementation of a perioperative protocol including RPRA and routine, planned ICU admission for high‐risk patients did not increase the hospital cost‐of‐care for EMAS [7]. There have been no Australian studies to date investigating the cost effectiveness of multidisciplinary perioperative protocols for EMAS such as ANZELA.

The ANZELA protocol was implemented as standard‐of‐care for EMAS patients at Bendigo Health in September 2021. This included RPRA and routine referral of ‘high‐risk’ patients to ICU for consideration of planned postoperative admission. As a regional centre with limited ICU resources, ICU admission is determined by the ICU team on a case‐by‐case basis. A retrospective audit found that following the introduction of ANZELA, unplanned ICU admissions reduced from 9.1% to 3.5%, with no associated rise in planned ICU admissions, ICU length‐of‐stay, hospital length‐of‐stay, mortality, or severe postoperative complications [3]. This suggests that the protocol may be a cost‐effective intervention, but further economic analysis is required.

Therefore, this project aims to evaluate whether RPRA and routine referral of high‐risk patients to ICU for consideration of planned postoperative admission constitutes a cost‐effective intervention that improves patient outcomes at Bendigo Health.

## 2 Methods

### 2.1 Setting

Bendigo Health acts as a regional referral centre for north‐west Victoria, Australia. The total catchment population is approximately 350 thousand [8]. The hospital's facilities include 724 beds, 11 operating theatres, and an ICU.

### 2.2 Study Design

This project was reviewed by the Bendigo Health Research Office and received Ethics Approval on 25/06/2024 (Reference LNR VIC/109006/BH‐2024‐434 003(v1)).

A retrospective audit was conducted including all high‐risk adult patients who underwent EMAS at Bendigo Health between 1st September 2017 and 31st August 2023. Postoperative outcomes and costs‐of‐care were compared before and after the implementation of ANZELA on 1st September 2021. A cost‐effectiveness analysis was conducted from the perspective of the healthcare system. Cost calculations were based on a time horizon extending to acute hospital discharge.

### 2.3 Inclusion Criteria

EMAS patients were shortlisted according to the inclusion criteria reported in the second ANZELA summary report [1], including procedures via both open and minimally invasive approaches. Surgeries for uncomplicated appendicitis, uncomplicated inguinal hernia, cholecystitis, trauma, postoperative complications, purely diagnostic purposes, or where no cost data was available were excluded. Bowel resections for inguinal hernia or complicated appendicitis with phlegmon were included.

Shortlisted adult EMAS patients had their perioperative risk calculated based on preoperative characteristics using the ‘NELA calculator’. Patients were considered high‐risk and included in the study if their perioperative mortality risk estimate was 10% or higher.

### 2.4 Data Sources

Patients who underwent EMAS prior to the implementation of ANZELA were identified by reviewing digital medical records of EMAS against the ANZELA inclusion/exclusion criteria [1]. Patients undergoing surgery following the implementation of ANZELA were prospectively identified by the treating team as standard‐of‐care.

### 2.5 Clinical Outcomes

The following variables were manually extracted from the digital medical record:

- *Patient Characteristics*, including demographics, preoperative NELA score, and operation performed. For patients who underwent surgery on or after 1 September 2021, NELA score was calculated preoperatively and documented in the medical record. For patients who underwent surgery pre‐ANZELA, or for whom NELA was not calculated and/or documented preoperatively, NELA was calculated retrospectively based on preoperative data as documented in the medical record.
- *Intensive Care Utilisation* details, including whether patients were discussed with ICU preoperatively, seen by an ICU outreach team preoperatively or in recovery, ICU length‐of‐stay (LOS) and whether the index ICU admission was planned or unplanned. Admission to ICU directly from theatre or recovery following an operation was considered planned. Where a patient was initially admitted to the ward and then later admitted to ICU following deterioration or unplanned return to theatre, the ICU admission was considered unplanned.
- *Surgical Outcomes*, including unplanned returns to theatre, severe postoperative complications, and mortality. Outcomes were measured at acute hospital discharge or 30 days post‐operation, whichever was later. Postoperative complications were classified using the Clavien‐Dindo classification [9]. A Clavien‐Dindo score of 3 or higher was considered a ‘severe’ complication.

### 2.6 Cost‐of‐Care Data

Admission ‘true cost’ data was extracted from the ‘Power Performance Manager’ (PPM) system. The PPM system consolidates clinical and financial data from various healthcare systems to measure the ‘true cost’ of a healthcare episode [10]. To account for inflation, cost estimates were indexed to the equivalent value of the Australian Dollar in 2023 ($2023AUD) using Consumer Price Indices available from the Australian Bureau of Statistics for ‘Melbourne – Hospital and Medical Services’ [10]. *Postoperative* cost, including cost incurred on or after the day of the index operation, was used for analysis to eliminate prolonged pre‐surgery hospital stays confounding costing results.

### 2.7 Analysis

Statistical and cost effectiveness analysis was completed using Stata statistical software (version SE 17; StataCorp) and Python software (version 3.8.13; Python Software Foundation, Massachusetts, United States). Outcomes were compared pre‐and post‐introduction of ANZELA. The Chi square test (2 sided) was used to analyze categorical data. Two‐Sample T and Mann–Whitney *U* tests were used to analyze normally distributed and skewed continuous data, respectively. A *p* value of < 0.05 was considered statistically significant.

A cost effectiveness analysis was performed to estimate the additional cost required to achieve an improvement in surgical outcomes. The cost effectiveness of the protocol, as relating to each outcome of interest, was presented as an incremental cost effectiveness ratio (ICER). ICERs were generated to estimate the additional cost per severe complication avoided, cost per unplanned return to theatre avoided, and cost per mortality avoided.

ICERs were calculated by comparing the difference in outcome with the difference in cost between the pre and post‐ANZELA cohorts, using the equation ICER = (Ca−Cb)/ (Oa−Ob), where:

- Ca is the mean postoperative cost pre‐ANZELA
- Cb is the mean postoperative cost post‐ANZELA
- Oa is the outcome rate in the pre‐ANZELA cohort
- Ob is the outcome rate in the post‐ANZELA cohort

To estimate uncertainty in the ICER values, bootstrapping procedures with 1000 replications were performed. This approach generated an empirical distribution of cost and outcome values and generated 95% confidence intervals for each ICER value.

## 3 Results

### 3.1 Baseline Characteristics

A total of 800 patients were identified as undergoing EMAS during the study period, with 226 of these being considered ‘high‐risk’ (i.e., having NELA mortality estimate of ≥ 10%). 31 patients for whom the index operation was a postoperative complication and 4 patients with no costing data available were excluded from analysis.

Baseline characteristics are shown in Table 1. There was no difference in Age (*p* = 0.90), Sex (*p* = 0.96), American Society of Anaesthesia (ASA) Classification (*p* = 0.78) or procedure type (*p* = 0.91) between cohorts. Figure S1 shows there was a trend toward higher mean NELA score in the pre‐ANZELA cohort; however, this was not statistically significant (26.0 vs. 21.4, *p* = 0.15).

**TABLE 1:** Patient demographics and baseline characteristics.

	All	Pre ANZELA	Post ANZELA	*p*
	*n* = 191	*n* = 131	*n* = 60	
Age (Mean, SD)	75.94 (11.16)	75.87 (10.47)	76.08 (12.64)	**0.903** ^a^
Sex (*n*, [%])				
Male	96 (50.26)	66 (50.38)	30 (50)	**0.961** ^b^
Female	95 (49.74)	65 (49.62)	30 (50)	
ASA (*n*, [%])				
3E	54 (28.27)	35 (26.72)	19 (31.67)	**0.778** ^b^
4E	124 (64.92)	87 (66.41)	37 (61.67)	
5E	13 (6.81)	9 (6.87)	4 (6.67)	
Preoperative discussion with ICU team (*n*, [%])	130 (68.59)	78 (59.54)	52 (86.67)	**< 0.001** ^b^
Subsequent perioperative ICU outreach review	122 (93.85)	75 (96.15)	47 (90.38)	**0.180** ^b^
Procedure type (*n*, [%])				
Colon resection with anastomosis	19 (9.95)	14 (10.69)	5 (8.33)	**0.935** ^b^
Colon resection without anastomosis	53 (27.75)	37 (28.24)	16 (26.67)	
Small bowel resection with anastomosis	28 (14.65)	21 (16.03)	7 (11.67)	
Small bowel resection without anastomosis	7 (3.66)	5 (3.82)	2 (3.33)	
Small bowel obstruction without resection	39 (20.42)	27 (20.61)	12 (20.00)	
Upper gastrointestinal	23 (12.04)	13 (9.92)	10 (16.67)	
Sepsis control	5 (2.62)	3 (2.29)	2 (3.33)	
Exploratory	10 (5.26)	7 (5.34)	3 (5.00)	
Other	7 (3.66)	4 (3.05)	3 (5.00)	
NELA score				
Mean (SD)	24.58 (15.50)	26.03 (16.69)	21.41 (12.05)	**0.153** ^c^
Median (IQR)	19.2 (13–30.2)	19.8 (13.8–32.6)	17 (12.93–24.2)	
NELA score range (*n*, [%])				
10–19.9	101 (52.88)	66 (50.38)	35 (58.33)	**0.170** ^b^
20–29.9	41 (21.47)	27 (20.61)	14 (23.33)	
30–39.9	17 (8.90)	14 (10.69)	3 (5.00)	
40–49.9	14 (7.33)	8 (6.11)	6 (10.00)	
50–100	18 (9.42)	16 (12.21)	2 (3.33)	

*Note:* *p* Values were highlighted in bold. Significant *p* Values were emphasised in red font.

^a^ Calculated with Independent *T* test.

^b^ Calculated with Chi square test.

^c^ Calculated with Mann Whitney *U* test.

### 3.2 ICU Resource Utilization

Significantly more patients were discussed with the ICU team preoperatively following the introduction of ANZELA (59.54% pre vs. 86.67% post‐ANZELA, *p* < 0.01). Of the patients referred to ICU, 93.85% were reviewed by an ICU outreach team preoperatively or in recovery. There was a *reduction* in the planned ICU admission rate from 78.63% to 63.33% (*p* = 0.03). The unplanned ICU admission rate was 6.3%, with no significant change post‐ANZELA (*p* = 0.96). Patients admitted to ICU stayed an average of 4.43 days. There was no difference in the planned (*p* = 0.76), unplanned (*p* = 0.45) or total (*p* = 0.47) ICU LOS post‐ANZELA.

### 3.3 Surgical Outcomes

Following the introduction of ANZELA, there was a 10.9% reduction in the rate of unplanned returns to theatre (17.56% vs. 6.67%, *p* = 0.045) and a 16.83% reduction in the rate of severe postoperative complications (61.83% vs. 45%, *p* = 0.03). The characteristics of the severe postoperative complications are shown in Table S1. The 30‐day postoperative mortality rate was 19.37%, with no significant change post‐ANZELA (18.32% vs. 21.67%, *p* = 0.59).

### 3.4 Cost of Hospital Care

The mean postoperative cost of hospital care, indexed to $2023 AUD, was $52 338.78. Mean cost of care was $53 930.58 pre‐ANZELA and $48 863.34 post‐ANZELA, as shown in Figure S2. There was a mean saving of $5067.24 per patient post‐ANZELA; however, this difference was not statistically significant (*p* = 0.98).

### 3.5 Cost Effectiveness

Table 3 summarises the ICER calculations. ANZELA was more effective and less costly with respect to rates of unplanned returns to theatre and severe postoperative complications. Post‐ANZELA, Bendigo health on average saved $46 528.63 (95% CI −$137 427.28, $199 123.10) per unplanned return to theatre avoided, and saved $30 104.69 (95% CI −$59 118.81, $18 5911.46) per severe postoperative complication avoided.

With regards to mortality, there was no statistically significant difference in outcome or cost between cohorts. The ICER value suggests a saving of $151 432.02 (95% CI −$1 305 393.30, $1 080 584.09) per additional mortality encountered post‐ANZELA.

## 4 Discussion

### 4.1 Cost Effectiveness

This economic analysis suggests that overall, RPRA and routine referral of high‐risk EMAS patients to ICU may be a cost‐effective intervention which improves patient care. There was a significant reduction in the rate of unplanned returns to theatre and severe postoperative complications following high‐risk EMAS, with no significant change in postoperative cost‐of‐care. This is consistent with Eveleigh et al., who also demonstrated the introduction of a perioperative protocol involving RPRA and routine ICU referral did not increase cost‐of‐care for EMAS [7]. The ICER values demonstrated in Table 3 have large confidence intervals, suggesting uncertainty in the calculated values. This is likely due to the small sample size (*n* = 191). However, given we can conclude there was no significant difference in postoperative cost and a reduction in surgical morbidity, the uncertainty found in the ICER values does not exclude ANZELA as a cost‐effective intervention.

### 4.2 Surgical Outcomes

This study demonstrated a reduction in postoperative morbidity for high‐risk EMAS following the introduction of ANZELA. Unplanned returns to theatre and severe postoperative complications were both significantly lower post‐ANZELA. Table S1 summarises the severe postoperative complications according to Clavien‐Dindo severity and system of worst complication, highlighting a notable difference in the rate of patients experiencing multi‐organ failure. RPRA and multidisciplinary care planning may aid in selecting appropriate perioperative dispositions necessary to facilitate early identification of deterioration, thus preventing ‘failure to rescue’ of postoperative complications.

Table 2 demonstrates that higher NELA scores were associated with higher rates of severe postoperative complications and higher costs of care. A trend toward lower mean preoperative NELA score post‐ANZELA was identified, which may contribute to the reduction in severe postoperative complications and unplanned returns to theatre. However, this trend may reflect that RPRA is prompting more appropriate selection of operative candidates. Whilst ‘No‐Lap’ patients were not included in this study, a previous audit demonstrated a trend toward higher up‐front palliation post the introduction of RPRA, and that higher NELA score was predictive of higher likelihood of up‐front palliation [3]. Table 1 indicates the percentage of patients with extremely high NELA scores (≥ 50%) reduced from 12.21% to 3.33% post‐ANZELA. RPRA may have identified extremely high‐risk patients where EMAS was likely futile, prompting up‐front palliation. More selective operating would contribute to a reduction in the overall risk profile of operative patients, in turn reducing postoperative complications and costs of care.

**TABLE 2:** Outcomes following high risk emergency abdominal surgery pre and post introduction of ANZELA.

Outcome		All	Pre ANZELA	Post ANZELA	*p*
		*n* = 191	*n* = 131	*n* = 60	
ICU utilisation					
Reviewed by ICU outreach perioperatively		163 (85.34)	114 (87.02)	49 (81.67)	**0.944** ^a^
Planned ICU admission (*n*, %)		141 (73.82)	103 (78.63)	38 (63.33)	**0.026** ^a^
No planned admission		50 (26.18)	28 (21.37)	22 (36.67)	
	Perioperative^b^ review by ICU outreach team	22 (44)	11 (39.29)	11 (50)	**0.574** ^a^
Unplanned ICU admission (*n*, %)		12 (6.28)	8 (6.15)	4 (6.66)	**0.959** ^a^
ICU length of stay (Mean, SD)	Total	4.43 (5.38)	4.5 (6.01)	4.24 (3.10)	**0.474** ^c^
	Planned	4.16 (4.89)	4.20 (5.53)	4.03 (3.03)	**0.454** ^c^
	Unplanned	4 (2.91)	3.94 (2.51)	4.20 (4.44)	**0.762** ^c^
Postoperative outcomes	NELA score				
Unplanned return to theatre (*n*, %)	All	27 (14.14)	23 (17.56)	4 (6.67)	**0.04 5** ^a^
	10‐19.9	14 (13.86)	12 (18.18)	2 (5.71)	
	20–29.9	5 (12.20)	4 (14.81)	1 (7.14)	
	30–39.9	2 (11.76)	2 (14.29)	0 (0)	
	40–49.9	2 (14.29)	1 (12.5)	1 (16.67)	
	50–100	4 (22.22)	4 (25)	0 (0)	
Severe^d^ post‐operative complication (*n*, %)	All	108 (56.54)	81 (61.83)	27 (45%)	**0 .030** ^a^
	10–19.9	42 (41.58)	33 (50)	9 (25.71)	
	20–29.9	25 (60.98)	16 (59.26)	9 (64.29)	
	30–39.9	14 (82.35)	12 (85.71)	2 (66.67)	
	40–49.9	11 (78.57)	6 (75)	5 (83.33)	
	50–100	16 (88.89)	14 (87.5)	2 (100)	
30 day mortality (*n*, %)	All	37 (19.37)	24 (18.32)	13 (21.67)	**0.587** ^a^
	10–19.9	13 (12.87)	9 (13.64)	4 (11.43)	
	20–29.9	7 (17.07)	3 (11.11)	4 (28.57)	
	30–39.9	6 (35.29)	5 (35.71)	1 (33.33)	
	40–49.9	4 (28.57)	2 (25)	2 (33.33)	
	50–100	7 (38.89)	5 (31.25)	2 (100)	
Post‐operative cost [$2023AUD] (Mean, SD)	All	52 338.78 (47 870.15)	53 930.58 (54 128.80)	48 863.34 (30 098.03)	**0.983** ^c^
	10–19.9	44 814.70 (26 367.63)	45 518.93 (26 521.27)	43 486.73 (26 408.11)	
	20–29.9	53 417.67 (29 698.24)	47 619.04 (24 475.70)	64 600.73 (36 210.02)	
	30–39.9	71 011.95 (96 715.76)	76 292.65 (10 6311.44)	46 368.70 (16 173.12)	
	40–49.9	65 882.00 (96 789.74)	84 175.11 (12 6321.45)	41 491.19 (27 716.36)	
	50–100	63 930.37 (49 976.55)	64 590.31 (51 046.23)	58 650.88 (57 547.02)	

*Note:* *p* Values were highlighted in bold. Significant *p* Values were emphasised in red font.

^a^ Chi square test.

^b^ Reviewed by ICU outreach team preoperatively or in theatre recovery, prior to return to ward.

^c^ Mann Whitney *U* test.

^d^ Clavien Dindo classification ≥ 3.

**TABLE 3:** Incremental cost effectiveness ratios (ICERs).

	Cost per outcome avoided^a^	95% confidence interval
Unplanned return to theatre	46 528.63	−137 427.28, 199 123.10
Severe^b^ postoperative complication	30 104.68	−59 118.81, 185 911.46
Mortality	−151 439.10	−1 305 393.30, 1 080 584.09

^a^ Converted to $2023AUD.

^b^ Clavien Dindo Classification ≥ 3.

Regional hospitals can deliver excellent outcomes for high‐risk surgical patients. The 30‐day postoperative mortality rate of 19.7% did not significantly change post‐ANZELA but was lower than the mean predicted mortality of 24.6%. This was favorable when compared to ANZELA and Ho et al., which reported 23.8% [11] and 29.7% [12] mortality rates amongst high‐risk EMAS cohorts, respectively.

### 4.3 Cost‐of‐Care

This project highlights the immense cost‐of‐care for EMAS patients. The average postoperative cost‐of‐care ($52 338.78 per patient) is comparable to the average cost range estimated by Burmas et al. of $26 856 to $61 037. Of note, Burmas et al. included *all* EMAS patients, whilst this project only included *high‐risk* EMAS patients. The two most costly episodes cost $431 689 and $394 884 respectively. Both were associated with severe postoperative complications leading to multiple unplanned returns to theatre, unplanned ICU admissions, protracted ICU stays, and subsequent high costs‐of‐care. These cases demonstrate how reducing severe postoperative complications through careful care planning might result in higher value care for EMAS patients. The lack of significant change in the cost‐of‐care post‐ANZELA may be attributable to the observed reduction in severe postoperative complications.

### 4.4 ICU Utilisation

Careful discussion between ICU and surgical teams appears to aid in judicious use of limited ICU resources, improving patient outcomes without compromising cost of care. In contrast to Eveleigh et al. and ANZELA recommendations, this study demonstrated an unexpected reduction in the rate of planned ICU admissions for high‐risk EMAS post‐ANZELA, dropping from 78% to 63%. This result suggests that routine postoperative ICU *admission* does not necessarily account for the improvement seen in postoperative outcomes. This may instead be the result of multidisciplinary care planning, including preoperative discussion with and perioperative review by ICU. There was a 27% increase in the likelihood of preoperative care planning discussions occurring between the surgical and intensive care teams following ANZELA's introduction. ICU outreach utilization did not increase post‐ANZELA. 93.85% of patients discussed with ICU preoperatively and 44% of patients where a planned ICU admission was declined were seen by an ICU outreach team preoperatively or in recovery, with no change post‐ANZELA (*p* = 0.18 and *p* = 0.57 respectively).

More careful preoperative planning may improve patient outcomes following EMAS with less ICU resource utilization than routine ICU admission based on NELA score alone. Whilst the reduction in planned ICU admissions is counter to the recommendations of ANZELA [1], there was no consequential increase in unplanned ICU admissions, ICU LOS, ICU outreach utilization, or negative postoperative outcomes. This project therefore supports routine preoperative discussion between surgical and intensive care outreach teams to guide high‐value perioperative care for high‐risk EMAS patients.

### 4.5 Limitations

This study has several limitations. Outcomes and cost‐of‐care were not adjusted to account for the risk profile of the included patients. There was a trend toward a higher (although not significant) mean NELA score amongst the pre‐ANZELA cohort, which may have a confounding effect on postoperative outcomes and cost‐of‐care findings. However, as discussed, the reduction in mean NELA score post‐ANZELA may be a result of RPRA prompting higher rates of up‐front palliation. Future studies could include a cost‐effectiveness analysis of all patients assessed for surgery, including patients palliated up‐front. Another limitation is the disparity in the size of the cohorts. Only 2 years of data post‐ANZELA was available at the time of data collection. We plan to repeat the project at 4 years post‐ANZELA to assess whether results are consistent over time.

This study was limited to exploring cost‐effectiveness from the hospital perspective in relation to specific patient outcomes. Due to the retrospective nature of the project, exploring the patient perspective by estimating cost effectiveness in terms of Quality Adjusted Life Years (QALY) was not possible; but should be considered in future research. Finally, this project excluded EMAS patients where the index surgery was due to a postoperative complication of elective surgery. This was due to the inability to isolate the postoperative cost in the costing system, but these patients should be considered for inclusion in future economic EMAS studies.

## 5 Conclusion

This study suggests that introducing a multidisciplinary perioperative protocol to guide perioperative care for EMAS in a regional health service can improve postoperative outcomes while also being cost effective. Introduction of the ANZELA protocol, including RPRA and routine ICU referral for high‐risk EMAS patients, reduced the rate of severe postoperative complications and unplanned returns to theatre without increasing cost of care. This is likely due to improved preoperative multidisciplinary care planning, rather than routine postoperative ICU admission.

## Conflicts of Interest

The authors declare no conflicts of interest.

## Supporting information


**Figure S1:** Distribution of NELA scores.

**Figure S2:** Postoperative cost of care for high‐risk emergency major abdominal surgery.

**Table S1:** Characteristics of postoperative complications pre and post ANZELA.

## Data Availability

The data that support the findings of this study are available on request from the corresponding author. The data are not publicly available due to privacy or ethical restrictions.
