# Supplementary figures and images for: Cost Effectiveness of a Multidisciplinary Perioperative Protocol for High‐Risk Emergency Major Abdominal Surgery in a Regional Victorian Hospital
Source: ANZ J Surg. 2025 Aug 22;95(10):2066–72. doi: 10.1111/ans.70299 (PMC12571933; doi:10.1111/ans.70299)

Figure S1:  
Distribution of NELA Scores

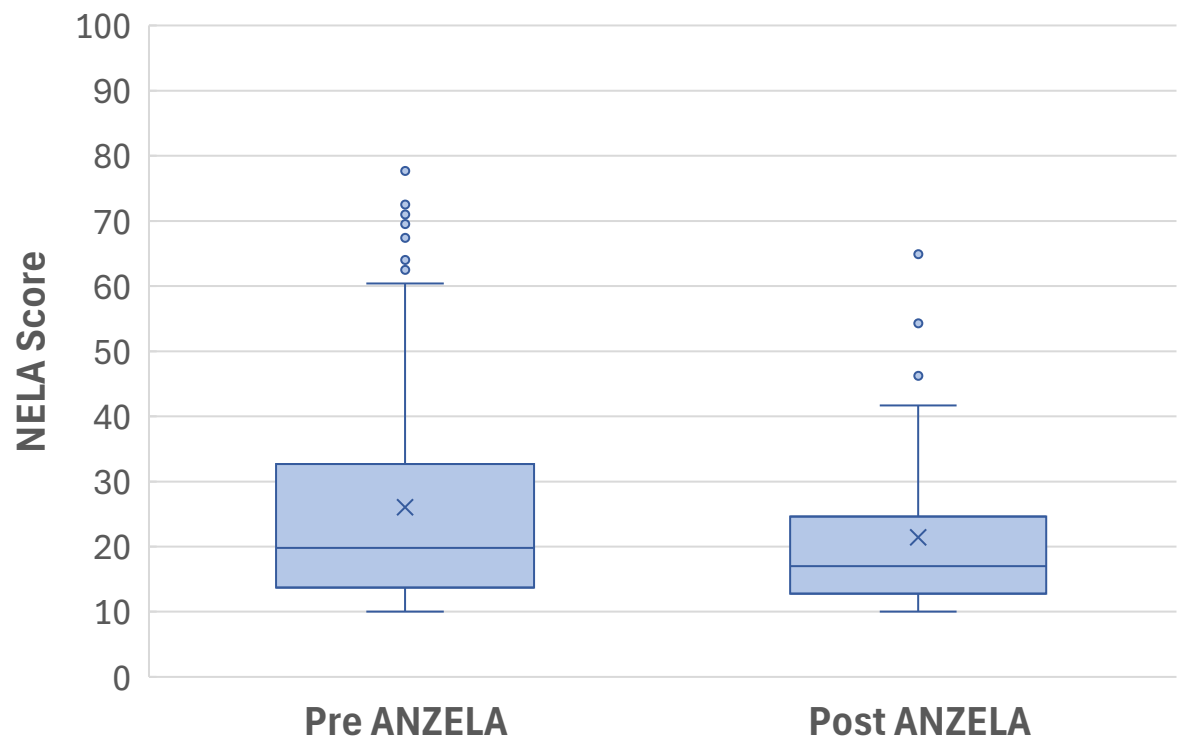

Supplement: Supplementary file 1 — Figure S1: Distribution of NELA scores. [file ANS-95-2066-s003.pdf]

**Figure S2 - Postoperative Cost of Care for High-risk  
Emergency Major Abdominal Surgery**

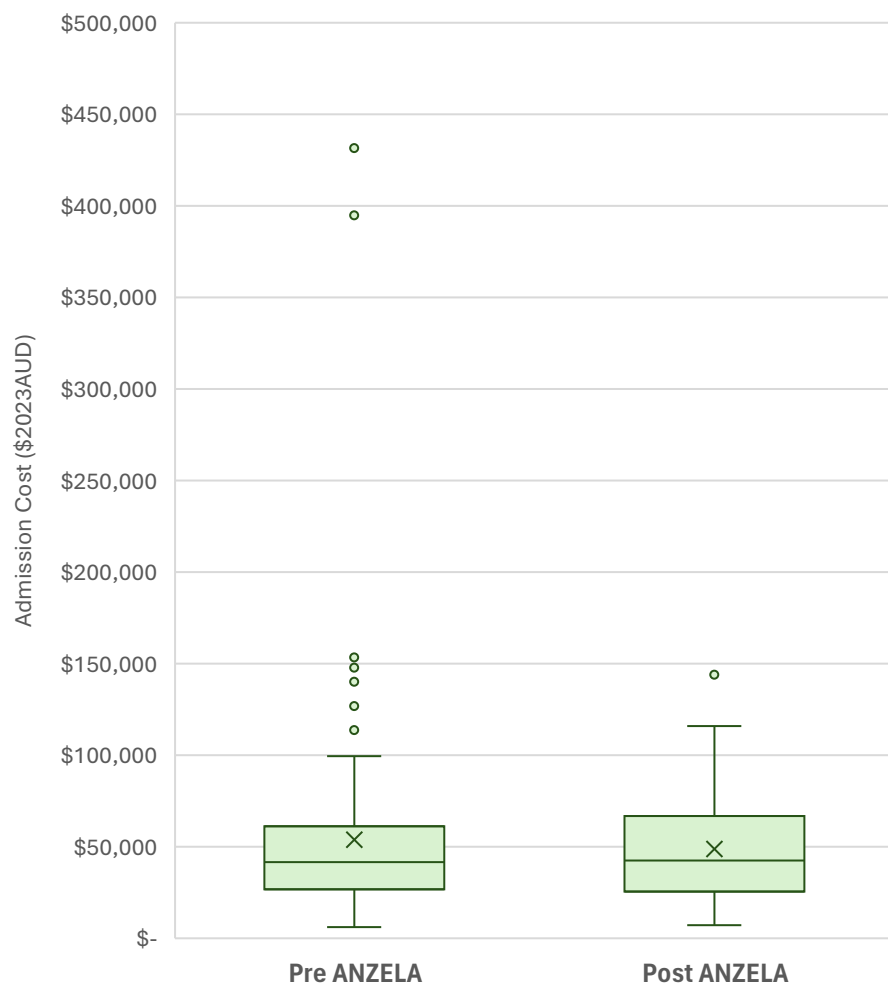

Supplement: Supplementary file 2 — Figure S2: Postoperative cost of care for high‐risk emergency major abdominal surgery. [file ANS-95-2066-s001.pdf]
