# Supplementary material for: Cost Effectiveness of a Multidisciplinary Perioperative Protocol for High‐Risk Emergency Major Abdominal Surgery in a Regional Victorian Hospital
Source: ANZ J Surg. 2025 Aug 22;95(10):2066–72. doi: 10.1111/ans.70299 (PMC12571933; doi:10.1111/ans.70299)
Supplement: Supplementary file 3 — Table S1: Characteristics of postoperative complications pre and post ANZELA. [file ANS-95-2066-s002.docx]

| **Table S1: Characteristics of Postoperative Complications Pre and Post ANZELA** | | | | |
| --- | --- | --- | --- | --- |
|  | **All** | **Pre ANZELA** | **Post ANZELA** | **P-value** |
| **Clavien Dindo Severity of Postoperative Complications** (n, %) | n=191 | n=131 | n=60 |  |
|  |  |  |  |  |
| 0 | 13 (6.81) | 4 (3.05) | 9 (15.00) | **0.003**^†^ |
| 1 | 24 (12.57) | 19 (14.50) | 5 (8.33) |  |
| 2 | 46 (24.08) | 27 (20.61) | 19 (31.67) |  |
| 3a | 1 (0.52) | 1 (0.76) | 0 (0.00) |  |
| 3b | 5 (2.62) | 3 (2.29) | 2 (3.33) |  |
| 4a | 51 (26.70) | 40 (30.53) | 11 (18.33) |  |
| 4b | 18 (9.42) | 17 (12.98) | 1 (1.67) |  |
| 5 | 33 (17.28) | 20 (15.27) | 13 (21.67) |  |
| **System of Severe Postoperative Complications** (n, %) | n=108 | n=81 | n=27 |  |
| Multi Organ Failure | 39 (20.42) | 32 (24.43) | 7 (11.67) | **0.194**^†^ |
| Cardiovascular | 44 (23.04) | 33 (25.19) | 11 (18.33) |  |
| Renal | 1 (0.52) | 1 (0.76) | 0 (0) |  |
| Respiratory | 9 (4.71) | 4 (3.05) | 5 (8.33) |  |
| Operative | 10 (5.24) | 8 (6.11) | 2 (3.33) |  |
| † - Calculated with Independent T Test | | | | |
